# Clinical Application of Pediatric Sleep Endoscopy: An International Survey

**DOI:** 10.3390/children11010094

**Published:** 2024-01-12

**Authors:** Giannicola Iannella, Giuseppe Magliulo, Antonio Greco, Armando De Virgilio, Antonino Maniaci, Jerome R. Lechien, Christian Calvo-Henriquez, Ahmed Yassin Bahgat, Manuele Casale, Rodolfo Lugo, Peter Baptista, Fabrizio Salamanca, Aurelio D’Ecclesia, Tiziano Perrone, Federico Leone, Angelo Cannavicci, Giovanni Cammaroto, Claudio Vicini, Annalisa Pace

**Affiliations:** 1 Department of Organi di Senso, Sapienza University, 00185 Rome, Italy; giuseppe.magliulo@uniroma1.it (G.M.); antonio.greco@uniroma1.it (A.G.); annalisa.pace@uniroma1.it (A.P.); 2 Department of Biomedical Sciences, Humanitas University, 20072 Milan, Italy; de_virgilio@hunimed.eu; 3 Department of Otolaryngology, Kore University, 94100 Enna, Italy; antonino.maniaci@unikore.it; 4 Department of Otolaryngology, Elsan Polyclinic of Poitiers, 86000 Poitiers, France; jerome.lechien@umons.ac.be; 5 Service of Otolaryngology, Rhinology Unit, Hospital Complex of Santiago de Compostela Travesía de Choupana, 15706 Santiago de Compostela, Spain; christian.ezequiel.calvo.henriquez@sergas.es; 6 Department of Otorhinolaryngology-Head & Neck Surgery, Alexandria University, Alexandria 5424041, Egypt; ahmedyassinbahgat@gmail.com; 7 Integrated Therapies in Otolaryngology, Fondazione Policlinico Universitario Campus Bio-Medico, 00128 Rome, Italy; m.casale@unicampus.it; 8 Department of Otorhinolaryngology, Grupo Medico San Pedro, Monterrey 64660, Mexico; rodo_lugo@me.com; 9 Department of Otorhinolaryngology, Clínica Universidad de Navarra, 31008 Pamplona, Spain; pmbaptista@unav.es; 10 Otorhinolaryngology Unit, San Pio X Hospital, 20159 Milan, Italy; fabrizio.salamanca@sanpiox.humanitas.it (F.S.); federico.leone@sanpiox.humanitas.it (F.L.); 11 IRCCS ‘Casa Sollievo della Sofferenza’, 71013 San Giovanni Rotondo, Italy; a.decclesia@operapadrepio.it; 12 Otorhinolaryngology Unit, Civil Hospital of Alghero, 07041 Alghero, Italy; tiziano.perrone@unife.it; 13 Head-Neck and Oral Surgery Unit, Department of Head-Neck Surgery, Otolaryngology, Morgagni Pierantoni Hospital, 47121 Forlì, Italy; angelo.cannavicci@auslromagna.it (A.C.); giovanni.cammaroto@auslromagna.it (G.C.); 14 Department ENT & Audiology, University of Ferrara, 44121 Ferrara, Italy; claudio.vicini@unife.it

**Keywords:** obstructive sleep apnea, drug-induced sleep endoscopy, pediatric OSA, pediatric DISE

## Abstract

Objectives: To investigate through an international survey the actual clinical application of drug-induced sleep endoscopy (DISE) in pediatric patients with obstructive sleep apnea (OSA) and to clarify the use, application, clinical indications, and protocol of pediatric DISE. Methods: A specific survey about pediatric DISE was initially developed by five international otolaryngologists with expertise in pediatric sleep apnea and drug-induced sleep endoscopy and was later spread to experts in the field of sleep apnea, members of different OSA-related associations. Results: A total of 101 participants who answered all the survey questions were considered in the study. Sixty-four sleep apnea experts, equivalent to 63.4% of interviewed experts, declared they would perform DISE in pediatric OSA patients. A total of 81.9% of responders agreed to consider the DISE as the first diagnostic step in children with persistent OSA after adenotonsillectomy surgery, whereas 55.4% disagreed with performing DISE at the same time of scheduled adenotonsillectomy surgery to identify other possible sites of collapse. In the case of young patients with residual OSA and only pharyngeal collapse during DISE, 51.8% of experts agreed with performing a velopharyngeal surgery. In this case, 27.7% disagreed and 21.4% were neutral. Conclusion: Pediatric DISE is internationally considered to be a safe and effective procedure for identifying sites of obstruction and collapse after adenotonsillectomy in children with residual OSA. This is also useful in cases of patients with craniofacial malformations, small tonsils, laryngomalacia or Down syndrome to identify the actual site(s) of collapse. Despite this evidence, our survey highlighted that pediatric DISE is not used in different sleep centers.

## 1. Introduction

Pediatric Obstructive Sleep Apnea (POSA) is defined by episodes of obstructed upper airways, either partially or entirely, recurring in children while they sleep. The prevalence of POSA is approximately 3% in children [1,2]. The pathophysiology of obstructive sleep apnea (OSA) disorder in children is multifactorial, but the two most significant risk factors are adenotonsillar hypertrophy and obesity [1,2,3,4]. Nevertheless, new evidence suggests that numerous children may experience a multilevel blockage in the upper airway while sleeping. This particularly applies to infants and children with underlying issues like craniofacial abnormalities, hypotonia, lingual tonsillar hypertrophy, and sleep state-dependent laryngomalacia [2,3,4]. Adenotonsillectomy is considered the first-line treatment modality for moderate to severe POSA children [4,5]. However, the possibility of a residual OSA after adenotonsillectomy has been reported in different clinical studies. The latest systematic review regarding this topic showed that the rate of residual disease (AHI > 5) in pediatric patients with a severe grade of OSA ranged from 30 to 55.5% [5,6,7,8,9,10]. 

Drug-induced sleep endoscopy (DISE) is a medical procedure designed to identify sites of upper airway obstruction in patients with sleep-related breathing disorders like OSA syndrome [1,2,3,4,5]. Performing an endoscopy during pharmacologically induced sleep enables the detection of upper airway obstructions and collapse, identifying their levels, types, and patterns. In adult OSA patients, it is a well-defined procedure that, in recent years, has been increasingly widespread, used, and accepted in the scientific context of sleep-breathing disorders [5,6,7,8,9,10].

The pediatric DISE in children with OSA is useful for identifying obstructive sites, especially in cases of residual OSAS after adenotonsillectomy surgery. In a recent clinical study, Mendes et al. identified, with a post-operative DISE, a multilevel obstruction in 11.3% of surgically treated patients with adenotonsillectomy. The authors suggested that in children with persistent OSA, the DISE can better characterize this subset of individuals and may guide toward an increasingly tailored surgery. In addition, a recent expert consensus statement regarding the management of pediatric persistent OSA after adenotonsillectomy, as agreed upon by the panel members, was that implementing DISE could potentially enhance PSG parameters in the treatment of persistent pediatric OSA. The evaluated parameters included AHI, the lowest oxygen saturation level (nadir), along with the quality of life and the burden of symptoms [1,2,3,4,5,6,7,8,9,10]. 

Despite this evidence, pediatric DISE is a less widespread procedure in sleep centers and is discussed more in the scientific literature compared to DISE in adult OSA patients [5,6,7,8,9,10,11,12,13,14,15]. 

Various reasons for the inferior use of such procedure could be related to some of its aspects: anesthesia-related risks, the complexity of pediatric airway anatomy, risk/benefit balance and lack of specialized training and expertise. Some authors have also stated a lower utility of this procedure in children and highlighted the possibility of a residual OSA even after DISE-directed surgery [6,13,14,15]. Therefore, nowadays, there are still many unresolved issues about the use and usefulness of pediatric DISE [1,2,3,4,5,6,7,8,9,10,11,12,13,14,15]. This study tries to fill in these knowledge gaps for a better understanding of pediatric DISE utility among otolaryngologists and sleep doctors. We designed an international survey in order to investigate the real clinical application of DISE in patients with POSA and to clarify the following controversial points:

– Use and application of pediatric DISE (primary outcome);
– Clinical indications for its use;
– How the DISE procedure is performed in children;
– Treatment choices for residual pediatric OSA according to the DISE outcomes.

## 2. Materials and Methods

### 2.1. Study Design and Setting

This research is a web-based cross-sectional international survey regarding the clinical spread of Pediatric DISE and its benefits in managing pediatric OSA patients. The study was designed at the sleep apnea center of the Organi di Senso Department of Sapienza University of Rome and shared with the sleep study group of the International Federation of Otorhinolaryngological Societies (YO-IFOS).

### 2.2. Survey

A specific survey about pediatric DISE was initially developed by five international otolaryngologists with certified expertise in sleep apnea, pediatric sleep apnea and drug-induced sleep endoscopy in adults and children. The web survey was created by using Google Survey (Mountain View, CA, USA), set in a way that each participant was allowed to fill in the survey just once.

The survey questions were based on a literature review focusing on pediatric DISE and embedded with the clinical and practical experience of the authors. The questions of the survey were structured in a way that experts could respond using a definite scale: disagree, neutral, or agree.

The survey’s aim was to investigate different aspects of pediatric DISE, such as clinical applications, use and utility, and interpretation of outcomes; therefore, the questions were divided into the following sub-items:

GENERAL QUESTIONS FOR SURVEY PARTICIPANTS: Medical doctor specialty, expertise to treat adult and/or pediatric OSA patients; expertise in performing adult and/or pediatric DISE; 

The participants who declared an absence of expertise in pediatric DISE were invited to stop the survey at these statements in order to obtain data only from experts on this topic in the subsequent specific sections. Obviously, among these participants, the debates that advocated against the use of this procedure were investigated.

PEDIATRIC DISE INDICATIONS: Eleven multiple choice questions regarding the use of pediatric DISE in different clinical POSA conditions.

DISE PROTOCOL: Eight multiple choice questions regarding how the pediatric DISE is performed by the OSA experts interviewed in the survey.

DISE OUTCOMES: Four multiple choice questions regarding the participant’s choice of treatment according to DISE evidence.

### 2.3. Participants of the Survey

The survey participants were identified from a pool of experts in the field of sleep apnea and were members of different OSA-related associations. All selected experts were qualified in pediatric OSA treatment. The survey was sent individually to all selected participants, and the answers were collected anonymously. The survey was conducted in July and August 2023. Incomplete responses were excluded from the analysis.

### 2.4. Ethical Considerations

The study received approval from the local Ethical Review Board of Sapienza University (code 6704), which stated that no additional ethical approval was necessary. Confidentiality was maintained regarding the questionnaire results, ensuring no linkages were established between these results and any particular surgeon. 

### 2.5. Statistical Analysis

Due to the investigative nature of the study, only a descriptive analysis of the study population was conducted.

## 3. Results

### 3.1. General Information

**Figure 1:**
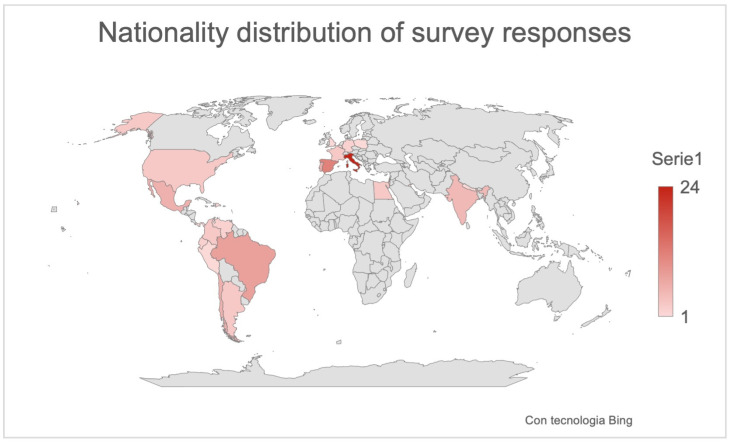
Global distribution of responses.

A total of 112 contacted sleep apnea experts were offered to take the survey, resulting in a 61% response rate. Eleven incomplete responses were disregarded in the analysis. A total of 101 participants who answered all the survey questions were considered in the study. The distribution of survey participants globally is shown in Figure 1.

Ninety-eight percent of responders were otolaryngologists, whereas the other two experts were one dentist (1%) and one pediatrician (1%). Figure 1 shows the global distribution of responses.

The participants’ experience in the field of OSAS and DISE for both adults and pediatric patients was investigated.

Ninety-nine percent of the interviewed experts declared that they would treat adult patients with OSAS, and 86.1% of these perform DISE in adult OSA patients. The same number of responders (96%) declared they also treated pediatric patients; however, in this case, only 64 sleep apnea experts, equivalent to 63.4% of those interviewed, noted that they perform DISE in pediatric OSA patients. The reasons they advocated against the use of DISE in children with OSA have been reported in Table 1.

### 3.2. Indication to the Pediatric DISE 

Questions concerning different possible clinical indications for pediatric DISE and how much the experts agreed or disagreed with these are reported in Table 2.

A total of 71.1% of experts advised against the use of pediatric DISE before any surgery to identify sites of collapse or possible multilevel obstruction, suggesting in case of POSA adenotonsillectomy surgery as the first line of treatment. The same majority of interviewed experts (55.4%) disagreed on performing DISE at the same time as scheduled adenotonsillectomy surgery to identify other possible sites of collapse. 

However, most experts agreed with the indication of performing a pediatric DISE before surgery in children with moderate–severe OSAS and small tonsils and adenoids (disproportion) (84.3% of respondents) and in children with Down syndrome or craniofacial abnormalities and OSA (65% of respondents).

Most of the experts agreed to consider a pediatric DISE in the diagnostic management of OSA children with suspected sleep-dependent laryngomalacia (74.6%) or muscular hypotonia (75.9%).

Finally, 81.9% of responders agreed to consider DISE as the first diagnostic step in children with persistent OSA after surgical adenotonsillectomy. Regarding this aspect, 79.2% of experts answered against the direct use of CPAP in case of AT-surgery failure.

**Figure 2:**
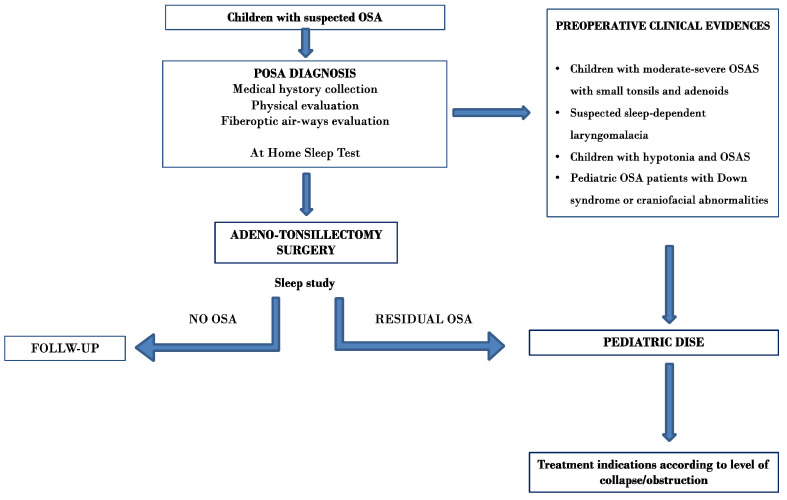
Potential algorithm for indicating pediatric DISE based on the survey results.

Figure 2 displays a flow chart illustrating a potential algorithm for indicating pediatric DISE based on the survey results.

### 3.3. DISE Protocol

Questions concerning the DISE protocol are reported in Table 3.

According to the experts’ opinion, propofol was the most frequent drug used when performing DISE in children (67.1%), followed by Dexmedetomidine (25.6%), Ketamine (4.9%) and Midazolam (2.4%).

VOTE classification was the most used scoring system during DISE (65.1%), followed by NOHL (15.7%), descriptive reports (15.7%) and the Chan–Parikh classification system (3.6%). 67.4% of interviewed experts used the Bispectral index (BIS) during the pediatric DISE.

Sixty-eight percent of experts agreed to the use of the ‘jaw thrust’ maneuver during pediatric DISE (57 experts). Likewise, 69.8% of experts agreed with the use of chin lift maneuvers. The majority of experts (73.4%) considered it useful to test the lateral position during the pediatric DISE, whereas the majority of respondents were indifferent (neutral) to testing the Head-Of-Bed Elevation (HOBE) during the pediatric DISE. Finally, 61.4% of experts answered “disagree” regarding the use of topical nasal decongestants to facilitate the advancement of the endoscope.

### 3.4. Patient’s Indications According to Pediatric DISE Outcomes

Questions and choice of eligible treatment according to DISE evidence are shown in Table 4. 

In the case of base-of-tongue anteroposterior collapse during DISE in a child with a previous adenotonsillectomy, 51.8% agreed with performing a base-of-tongue resective or ablative surgery, whereas 22.8% were against this surgical approach and 19.3% were neutral. In cases of children with severe OSA and only collapses of the epiglottis during DISE, 71% of experts agreed with performing an epiglottis surgery. Accordingly, in the case of young patients with residual OSA and only pharyngeal collapse during DISE, 51.8% of experts stated they agreed with velopharyngeal surgery; however, 27.7% disagreed, and 21.4% were neutral.

## 4. Discussion

In recent years, the use of DISE has gained popularity as a method for assessing the dynamic blockages in the upper airways that occur during sleep and guiding decisions regarding the treatment of OSA patients. Sleep surgeons globally using DISE and the notable progress in DISE-related studies have resulted in a growing body of literature agreeing on the effectiveness and application of DISE for adult OSA patients [15,16,17,18,19,20,21]. 

On the other hand, the attention towards including DISE in the treatment regimen for pediatric OSA has emerged relatively recently and slowly. In addition, the available evidence showcasing its usefulness and efficiency is insufficient. The majority of literature regarding this subject is constituted by small case series or retrospective studies [17,18,19,20,21,22,23,24,25,26,27,28]. Despite evidence demonstrating that DISE impacts the decisions made regarding surgery in children, it remains unclear how many sleep doctors actually use this procedure and if the results from interventions guided by DISE surpass those from conventional methods. The areas of debate involve the appropriate indications, best sedation approach, endoscopy procedure protocol, and understanding DISE discoveries. With these gaps in understanding about DISE’s role in pediatric OSA management, we selected this topic for an international survey of OSA experts. The objective of this survey was to investigate expert opinions in relation to employing pediatric DISE and the disputed aspects to minimize variations in practice and enhance the quality of care for pediatric OSA patients [5,6,7,8,9,10,11,12].

Of the 101 sleep apnea experts participating in the survey who answered all the survey questions, only 63.4% reported they performed DISE in pediatric OSA patients. In our opinion, this percentage reflects and clarifies the true distribution of pediatric DISE use worldwide. 

In relation to the question of the reasons why many sleep doctors do not perform this procedure, we investigated this aspect and clarified the different reasons the experts provided against the use of this procedure in pediatric OSA patients:

Lack of Experience and Training: Pediatric DISE requires specialized training and expertise due to the unique considerations when dealing with children. The medical sleep community might need more awareness and education regarding the real benefits and appropriate use of pediatric DISE.

Anesthesia Risks in Pediatrics: Administering sedation or anesthesia to children involves specific expertise and risks. Pediatric patients can have various responses to sedation, and ensuring their safety during the procedure is a primary aspect. The absence of a pediatric anesthesiologist team with expertise in children’s sedation is crucial when performing this procedure accurately and safely.

Ethical Considerations: Performing procedures on pediatric patients requires careful considerations of the real need for them. It is important to weigh the potential benefits of the procedure versus its risks. Informed consent from parents or guardians is crucial.

Limited Data and Guidelines: Compared to adults, there might be a scarcity of comprehensive data on prospective randomized studies and a lack of established guidelines for pediatric DISE. 

Resource Constraints: Performing pediatric DISE might require specific equipment, facilities and a multidisciplinary team, which could be limited in some medical centers.

Complexity of Pediatric Airway Anatomy: Pediatric airway anatomy is different from that of adults, and the causes of upper airway obstruction can vary widely. This complexity might interpret DISE findings more challenging in children.

Low uptake: Some experts considered this procedure useful in some selected neurological or craniofacial deformities cases in children with OSAS.

Expensive: The high ratio between the cost and reimbursements of this procedure makes it economically unfavorable compared to other non-invasive diagnostic procedures.

### 4.1. Indication to the Pediatric DISE 

The first cause of pediatric OSA is upper airway obstruction, which is related to adenotonsillar hypertrophy. 

Adenotonsillectomy is currently recommended by both the American Academy of Otolaryngology Head and Neck Surgery as well as the American Academy of Pediatrics as a first-line treatment for children with OSA documented by night polysomnography, with a success rate of approximately 80% [1,5,6,7,8,9,10,11,12,13,14,15].

In a systematic review, Venekamp RP et al. [29] compared tonsillectomy or adenotonsillectomy versus non-surgical management for obstructive sleep-disordered breathing in children. The authors concluded that in healthy children and those diagnosed with mild to moderate OSAS by PSG, there was high-quality evidence that this procedure shows benefits concerning PSG parameter improvement and moderate-quality evidence that A-T surgery provides a benefit in terms of children’s behavior, their quality of life and their symptoms.

In our survey, 71.1% of the experts advised against the use of pediatric DISE before A-T surgery to identify sites of collapse or possible multilevel obstruction in children with naïve diagnosis of OSA. The same majority of surveyed experts (55.4%) disagreed with performing DISE at the same time as scheduled adenotonsillectomy surgery to identify other possible sites of collapse. 

These data confirm most of the evidence in the literature, suggesting that the first line of treatment for healthy pediatric OSA should be surgical adenotonsillectomy [15,16,17,18,19,20,21,22,23,24,25]. 

Most experts agreed with the indication of performing a pediatric DISE before surgery in children with moderate–severe OSAS and small tonsils and adenoids (disproportion) (84.3% of respondents) and in children with Down syndrome or craniofacial abnormalities and OSA (65% of respondents). In addition, 81.9% of responders agreed to consider DISE the first diagnostic step in children with persistent OSA after adenotonsillectomy surgery. Regarding this aspect, 79.2% of experts disagreed with the direct use of CPAP in cases of AT-surgery failure.

These data align with the expert consensus statement on pediatric DISE [1]. The authors of this international study reported that DISE was recommended for the following scenarios: (1) children having OSA with small tonsils, (2) children experiencing ongoing OSA after adenotonsillectomy, and (3) during A-T surgery of pediatric patients with an elevated risk of persistent OSA. 

The panel of experts highlighted that when targeted, upper airway surgery for ongoing OSA can be both safe and efficient. The importance has been acknowledged involving the direction of surgery towards specific sites of obstruction identified during the diagnostic workup with DISE following the AT operation for children with persistent OSA.

### 4.2. DISE Protocol

A thorough retrospective analysis of the literature regarding anesthetic plans for pediatric DISE was recently conducted by Liu et al. [23]. The authors reported that propofol emerged as the most frequently utilized anesthetic. Among the studies, three employed a blend of remifentanil and propofol, one exclusively used dexmedetomidine, another applied only sevoflurane, and one study contrasted various protocols. Dexmedetomidine and ketamine present the most promising characteristics for pediatric DISE but aren’t uniformly adopted. According to the expert opinion of our survey, Propofol was the most frequent drug used when performing pediatric DISE (67.1%), followed by Dexmedetomidine (25.6%), Ketamine (4.9%) and Midazolam (2.4%).

One of the key aspects when performing DISE is how to report its results. Several classification systems have been proposed for this purpose in adults and children [5,6,7,8,9,10,11,12,13,14,15]. Each of these assesses and reports sites of obstruction and collapse differently.

The VOTE classification was the most used scoring system, which emerged on our survey (65.1%), followed by NOHL (15.7%), descriptive report (15.7%) and the Chan—Parikh classification system (3.6%). This data seems to agree with the data available in the literature. Amos et al. [30], in a systematic review of drug-induced sleep endoscopy scoring systems used in children, reported that the VOTE system was the most common scoring system documented for children undergoing DISE; however, there was no consensus concerning the selection of the scoring system to report observations during DISE.

#### Patient’s Indications According to Pediatric DISE Outcomes

Could tongue-base surgery be a valid treatment for treating children with tongue-base collapse?

In the case of base-of-tongue anteroposterior collapse during DISE in a child with previous adenotonsillectomy, 51.8% of experts noted in the survey stated they agreed with performing base-of-tongue resective or ablative surgery. This data is in agreement with the evidence in the literature. In a case series described by Williamson et al. [15], a lingual tonsillectomy or a midline posterior glossectomy was chosen as the treatment of OSA in pediatric patients with base-of-tongue collapse evidenced by DISE. A total of 168 children, with a mean ± SD age of 8.3 ± 3.6 years, were part of the study. Among them, 101 underwent lingual tonsillectomy alone, 25 had midline posterior glossectomy alone, and 42 received both procedures. The mean improvement in AHI was 3.52 ± 8.39, 2.55 ± 5.59, and 3.70 ± 6.07, respectively. All surgical groups showed significant benefits in sleep apnea parameters based on a comparison of pre- and postoperative AHI (*p* < 0.01). Overall surgical success, defined by an AHI < 1 (or <5 without clinical symptoms), was achieved in 75% (126 patients). Camacho et al. [31] conducted a meta-analysis study of 114 patients who underwent surgeries targeting pre- and post-BOT reduction, which resulted in a reduction of the apnea-hypopnea index (AHI) from a mean (M) and standard deviation (SD) of 16.9 ± 12.2/h to 8.7 ± 10.6/h (48.5% decrease). The authors concluded that when directed by drug-induced sleep endoscopy, surgery focusing on the pediatric tongue base shows notable potential in significantly enhancing AHI, indicating promising success in addressing base-of-tongue collapse among pediatric obstructive sleep apnea patients.

In cases of children with severe OSA and only a collapse of the epiglottis during DISE, 71% of the experts agreed with performing an epiglottis surgery. Good outcomes of supraglottoplasty for POSA have been reported in a meta-analysis by Lee CF et al. [32]. Their analysis encompassed eleven studies involving 121 patients (average age: 3.7 years; 64% male; mean sample size: 11 patients). Following surgery, significant improvements were observed in the difference between pre- and postoperative measurements, showing an 8.9 events/h reduction in the AHI and a 3.7% increase in minimum oxygen saturation (MinSaO2; *p* < 0.05). The overall success rate post-surgery was 28% based on an AHI < 1 and 72% based on an AHI < 5. Therefore, as also reported in the expert consensus statement about the management of pediatric persistent obstructive sleep apnea, supraglottoplasty proves to be a secure and efficient remedy for children experiencing ongoing OSA linked to sleep-dependent laryngomalacia identified during DISE.

In cases of young patients with residual OSA and only pharyngeal collapse during DISE, a pharyngoplasty surgery has been proposed by different authors with good results compared with tonsillectomy alone or in children with residual pharyngeal collapse after A-T surgery. In our study, 51.8% of experts agreed with performing a velopharyngeal surgery; however, in this case, 27.7% disagreed, and 21.4% were neutral. According to the expert consensus statement on pediatric persistent obstructive sleep apnea post-adenotonsillectomy management, expansion pharyngoplasty proves to be a secure and effective remedy for pediatric patients facing persistent OSA associated with lateral wall collapse identified during DISE [1,33].

One limitation of the study was investigating a population of experts that does not reflect the world’s geographical distribution. This limitation arose from the survey’s distribution limited to the European and South American Sleep Medicine Societies.

## 5. Conclusions

Pediatric DISE is considered internationally to be a safe and effective procedure for identifying sites of obstruction and collapse after adenotonsillectomy in children with residual OSA. It is also useful in cases of patients with craniofacial malformations, small tonsils, laryngomalacia or Down syndrome to identify the real site(s) of collapse. Despite this evidence, our survey highlighted that pediatric DISE is not used in various sleep centers.

## Figures and Tables

**Table 1:** Distribution of expert-advocated reasons against the use of the pediatric DISE.

Advocated Reasons against the Use of the Pediatric DISE in OSA Children of 37 Participants to the Survey	Number	Percentage
Lack of pediatric DISE experience at the sleep center	10	27%
Absence of an anesthesiologic pediatric team with expertise in children’s sedation—Anesthesia Risks	7	18.9%
Not useful procedures	9	24.3%
Procedure useful in very selected cases (neurological cases—craniofacial deformities children)	5	13.5%
Not favorable risk/benefit ratio	5	13.5%
Expensive	1	2.7%

**Table 2:** Experts’ responses of clinical indications to the pediatric DISE.

PEDIATRIC DISE INDICATIONS—83 Participants	Disagree	Neutral	Agree
In cases of healthy pediatric patients with a new diagnosis of OSA (At-Home Sleep Test), do you consider DISE as the first test before adenotonsillectomy surgery in order to identify sites of collapse?	59 (71%)	15 (18.1%)	9 (10.8%)
In cases of healthy pediatric patients with a new diagnosis of OSA (At-Home Sleep Test), do you consider DISE at the same time as scheduling adenotonsillectomy surgery to identify other possible sites of collapse?	46 (55.4%)	10 (12%)	27 (32.5%)
In the case of children with moderate–severe OSAS with small tonsils and adenoids (disproportion), do you consider DISE indicated before surgery?	11 (13.2%)	2 (2.4%)	70 (84.3%)
Do you consider a DISE as a first-line test in cases of suspected sleep-dependent laryngomalacia?	15 (18%)	6 (7.2%)	62 (74.6%)
In children with hypotonia and OSAS, do you consider DISE in the diagnostic management?	7 (8.4%)	13 (16.9%)	63 (75.9%)
In cases of patients with Down syndrome or craniofacial abnormalities and OSA, do you consider DISE before surgery?	11 (13.3%)	18 (21.7%)	54 (65%)
In cases of persistent OSA in children after adenotonsillectomy, do you suggest DISE as the first diagnostic step?	10 (12%)	5 (6%)	68 (81.9%)
In cases of persistent OSA in children after adenotonsillectomy, do you consider CPAP or other treatments as a first line treatment avoiding DISE?	65 (78.3%)	12 (14.6%)	5 (6%)

**Table 3:** Expert responses related to pediatric DISE protocol.

PEDIATRIC DISE PROTOCOL—83 Participants	Disagree	Neutral	Agree
Do you consider maneuvers such as jaw thrusts useful during pediatric DISE?	7 (8.4%)	19 (22.9%)	57 (68.6%)
Do you consider maneuvers such as chin lifts useful during pediatric DISE?	4 (6.8%)	21 (25.3%)	58 (69.8%)
Do you consider testing the lateral position during the pediatric DISE?	6 (7.2%)	16 (19.3%)	61 (73.4%)
Do you consider testing the Head-Of-Bed Elevation (HOBE) during the pediatric DISE?	8 (9.6%)	39 (46.9%)	36 (43.3%)
Do you consider the use of topical nasal decongestants in order to facilitate the advancement of the endoscope?	51 (61.4%)	15 (18.1%)	17 (20.4%)

**Table 4:** Experts’ responses of treatment indications according to the pediatric DISE.

TREATMENT INDICATIONS ACCORDING TO PEDIATRIC DISE OUTCOMES—83 Participants	Disagree	Neutral	Agree
In cases of base-of-tongue anteroposterior collapse during DISE in children with previous adenotonsillectomy surgery, do you consider base-of-tongue surgery?	19 (22.8%)	16 (19.3%)	48 (57.8%)
In cases of children with severe OSA, comorbidities or craniofacial malformations and only the collapse of big tonsils during DISE, do you consider only the tonsillectomy?	6 (7.2%)	4 (5.8%)	73 (87.9%)
In cases of young patients with severe OSA and only epiglottis collapses during DISE, do you consider epiglottis surgery?	10 (12%)	15 (17.9%)	59 (71%)
In cases of young patients with residual OSA and only pharyngeal collapse during DISE, do you consider velopharyngeal surgery?	23 (27.7%)	18 (21.4%)	43 (51.8%)

## Data Availability

The data presented in this study are available on request from the corresponding author. Data are unavailable due to privacy or ethical restrictions.

## References

[1] Parikh S.R., Boudewyns A., Friedman N.R., Schwartz S.R., Baldassari C.M., Benedek P., Carr M.M., Chan D.K., Chan K.C., Cheng A.T. (2023). International Pediatric Otolaryngology Group (IPOG) consensus on scoring of pediatric Drug Induced Sleep Endoscopy (DISE). Int. J. Pediatr. Otorhinolaryngol..

[2] Cielo C.M., Tapia I.E. (2023). What’s New in Pediatric Obstructive Sleep Apnea?. Sleep Med. Clin..

[3] Mussi N., Forestiero R., Zambelli G., Rossi L., Caramia M.R., Fainardi V., Esposito S. (2023). The First-Line Approach in Children with Obstructive Sleep Apnea Syndrome (OSA). J. Clin. Med..

[4] Qarbal J., Le Treut-Gay C., Allali L., Rossi M.E., Nicollas R., Moreddu E. (2023). Drug-Induced sleep endoscopy in children: NAVOTEL scoring system development. Pediatr. Pulmonol..

[5] Mendes N., Antunes J., Guimarães A., Adónis C., Freire F. (2023). Severe Pediatric Sleep Apnea: Drug-Induced Sleep Endoscopy Based Surgery. Indian. J. Otolaryngol. Head Neck Surg..

[6] Shman S.L., Maturo S., Schwartz S., McKenna M., Baldassari C.M., Bergeron M., Chernobilsky B., Ehsan Z., Gagnon L., Liu Y.C. (2023). Expert Consensus Statement: Management of Pediatric Persistent Obstructive Sleep Apnea After Adenotonsillectomy. Otolaryngol. Head Neck Surg..

[7] Iannella G., Magliulo G., Cammaroto G., Meccariello G., De Vito A., Pelucchi S., Ciorba A., Maniaci A., Cocuzza S., Gulotta G. (2022). Effectiveness of drug-induced sleep endoscopy in improving outcomes of barbed pharyngoplasty for obstructive sleep apnea surgery: A prospective randomized trial. Sleep Breath..

[8] Borrelli M., Corcione A., Rongo R., Cantone E., Scala I., Bruzzese D., Martina S., Strisciuglio P., Michelotti A., Santamaria F. (2022). Obstructive Sleep Apnoea in Children with Down Syndrome: A Multidisciplinary Approach. J. Pers. Med..

[9] Castillo-García M., Solano-Pérez E., Coso C., Romero-Peralta S., García-Borreguero D., Izquierdo J.L., Mediano O. (2023). Impact of obstructive sleep apnea in cardiovascular risk in the pediatric population: A systematic review. Sleep Med. Rev..

[10] Frederick R.M., Brandt J., Sheyn A. (2022). Drug-induced sleep endoscopy effect on intraoperative decision making in pediatric sleep surgery: A 2-year follow up. Laryngoscope Investig. Otolaryngol..

[11] Kirkham E.M. (2022). Pediatric Drug-Induced Sleep Endoscopy. Otolaryngol. Clin. N. Am..

[12] Esteller Moré E., Navazo Egía A.I., Carrasco Llatas M. (2020). Drug-induced sleep endoscopy in children. Acta Otorrinolaringol. (Engl. Ed.).

[13] Sharma A.V., Padhya T., Nallu S. (2022). Management of Pediatric Obstructive Sleep Apnea After Failed Tonsillectomy and Adenoidectomy. Adv. Pediatr..

[14] Li C., Kou Y.F., DeMarcantonio M.A., Heubi C.H., Fleck R., Kandil A., Smith D.F., Ishman S.L. (2023). Sleep Endoscopy and Cine Magnetic Resonance Imaging Evaluation of Children with Persistent Obstructive Sleep Apnea. Otolaryngol. Head Neck Surg..

[15] Williamson A., McArdle E.H., Morrow V.R., Zalzal H.G., Carr M.M., Coutras S.W. (2023). Base of Tongue Surgery and Pediatric Obstructive Sleep Apnea. Otolaryngol. Head Neck Surg..

[16] Williamson A., Coutras S.W., Carr M.M. (2022). Sleep Endoscopy Findings in Children with Obstructive Sleep Apnea and Small Tonsils. Ann. Otol. Rhinol. Laryngol..

[17] Kang K.T., Hsu W.C. (2023). Efficacy of adenotonsillectomy on pediatric obstructive sleep apnea and related outcomes: A narrative review of current evidence. J. Formos. Med. Assoc..

[18] Cousineau J., Prévost A.S., Battista M.C., Gervais M. (2021). Management of obstructive sleep apnea in children: A Canada-wide survey. J. Otolaryngol. Head Neck Surg..

[19] Arganbright J.M., Lee J.C., Weatherly R.A. (2021). Pediatric drug-induced sleep endoscopy: An updated review of the literature. World J. Otorhinolaryngol. Head Neck Surg..

[20] Baldassari C.M., Lam D.J., Ishman S.L., Chernobilsky B., Friedman N.R., Giordano T., Lawlor C., Mitchell R.B., Nardone H., Ruda J. (2021). Expert Consensus Statement: Pediatric Drug-Induced Sleep Endoscopy. Otolaryngol. Head Neck Surg..

[21] Ergenekon A.P., Gokdemir Y., Ersu R. (2023). Medical Treatment of Obstructive Sleep Apnea in Children. J. Clin. Med..

[22] Hyzer J.M., Milczuk H.A., Macarthur C.J., King E.F., Quintanilla-Dieck L., Lam D.J. (2021). Drug-Induced Sleep Endoscopy Findings in Children with Obstructive Sleep Apnea with vs without Obesity or Down Syndrome. JAMA Otolaryngol. Head Neck Surg..

[23] Liu K.A., Liu C.C., Alex G., Szmuk P., Mitchell R.B. (2020). Anesthetic management of children undergoing drug-induced sleep endoscopy: A retrospective review. Int. J. Pediatr. Otorhinolaryngol..

[24] Love H., Slaven J.E., Mitchell R.M., Bandyopadhyay A. (2020). Outcomes of OSA in surgically naïve young children with and without DISE identified laryngomalacia. Int. J. Pediatr. Otorhinolaryngol..

[25] Kirkham E.M., Hoi K., Melendez J.B., Henderson L.M., Leis A.M., Puglia M.P., Chervin R.D. (2021). Propofol versus dexmedetomidine during drug-induced sleep endoscopy (DISE) for pediatric obstructive sleep apnea. Sleep Breath..

[26] Raposo D., Menezes M., Rito J., Trindade-Soares M., Adónis C., Loureiro H.C., Freire F. (2021). Drug-Induced Sleep Endoscopy in Pediatric Obstructive Sleep Apnea. Otolaryngol. Head Neck Surg..

[27] Kirkham E.M., Melendez J.B., Hoi K., Chervin R.D. (2021). Drug-Induced Sleep Endoscopy in Children with Positional Obstructive Sleep Apnea. Otolaryngol. Head Neck Surg..

[28] Smith D.F., He S., Peddireddy N.S., Vairavan Manickam P., Heubi C.H., Shott S.R., Cohen A.P., Ishman S.L. (2020). Effectiveness of pediatric drug-induced sleep endoscopy for REM-predominant obstructive sleep apnea. Sleep Breath..

[29] Venekamp R.P., Hearne B.J., Chandrasekharan D., Blackshaw H., Lim J., Schilder A.G. (2015). Tonsillectomy or adenotonsillectomy versus non-surgical management for obstructive sleep-disordered breathing in children. Cochrane Database Syst. Rev..

[30] Amos J.M., Durr M.L., Nardone H.C., Baldassari C.M., Duggins A., Ishman S.L. (2018). Systematic Review of Drug-Induced Sleep Endoscopy Scoring Systems. Otolaryngol. Head Neck Surg..

[31] Camacho M., Noller M.W., Zaghi S., Reckley L.K., Fernandez-Salvador C., Ho E., Dunn B., Chan D. (2017). Tongue surgeries for pediatric obstructive sleep apnea: A systematic review and meta-analysis. Eur. Arch. Otorhinolaryngol..

[32] Lee C.F., Hsu W.C., Lee C.H., Lin M.T., Kang K.T. (2016). Treatment outcomes of supraglottoplasty for pediatric obstructive sleep apnea: A meta-analysis. Int. J. Pediatr. Otorhinolaryngol..

[33] Iannella G., Cammaroto G., Meccariello G., Cannavicci A., Gobbi R., Lechien J.R., Calvo-Henríquez C., Bahgat A., Di Prinzio G., Cerritelli L. (2022). Head-of-Bed Elevation (HOBE) for Improving Positional Obstructive Sleep Apnea (POSA): An Experimental Study. J. Clin. Med..

